# Heme Uptake and Utilization by Gram-Negative Bacterial Pathogens

**DOI:** 10.3389/fcimb.2019.00081

**Published:** 2019-03-29

**Authors:** Kaylie L. Richard, Brittni R. Kelley, Jeremiah G. Johnson

**Affiliations:** Department of Microbiology, University of Tennessee, Knoxville, TN, United States

**Keywords:** iron, heme, hemolysins, heme oxygenase, hemophores, heme transport

## Abstract

Iron is a transition metal utilized by nearly all forms of life for essential cellular processes, such as DNA synthesis and cellular respiration. During infection by bacterial pathogens, the host utilizes various strategies to sequester iron in a process termed, nutritional immunity. To circumvent these defenses, Gram-negative pathogens have evolved numerous mechanisms to obtain iron from heme. In this review we outline the systems that exist in several Gram-negative pathogens that are associated with heme transport and utilization, beginning with hemolysis and concluding with heme degradation. In addition, Gram-negative pathogens must also closely regulate the intracellular concentrations of iron and heme, since high levels of iron can lead to the generation of toxic reactive oxygen species. As such, we also provide several examples of regulatory pathways that control heme utilization, showing that co-regulation with other cellular processes is complex and often not completely understood.

## Introduction

Iron is a transition metal that is essential for Gram-negative pathogen growth and successful colonization of a host (Palmer and Skaar, 2016; Sheldon et al., 2016). This requirement of iron is due to its use as a cofactor for several cellular processes in both the host and pathogen, such as cellular respiration (Meneghetti et al., 2016) and DNA synthesis (Hood and Skaar, 2012). Although necessary for growth, iron can become toxic when concentrations are too high (Andrews et al., 2003) since it acts as a reducing agent and aids in reactive oxygen species (ROS) generation. These reactive oxygen species can then damage intracellular molecules, like DNA, and affect cell viability (Touati, 2000; Andrews et al., 2003). Because of these detrimental effects, concentrations of iron need to be tightly controlled to reduce the potential for toxicity and damage.

During infection by pathogenic organisms, including Gram-negative bacteria, the availability of free-iron is targeted by the host immune response (Palmer and Skaar, 2016). Specifically, the host attempts to limit iron-availability to the invading pathogen through a process termed, nutritional immunity (Barber and Elde, 2014; Cornelissen, 2018), which results in free-iron levels as low as 10^−24^ M (Raymond et al., 2003). This restriction process typically involves the production of molecules that bind free-iron and prevent its uptake by pathogenic organisms, while keeping the iron available for use by the host (Barber and Elde, 2014).

The roles of various iron-binding molecules during infection have been demonstrated in multiple hosts for several pathogens (Jordan and Saunders, 2009; Nasioudis and Witkin, 2015; Vazquez-Gutierrez et al., 2016). For example, host lipocalin-2 was shown to reduce the growth of pathogenic *E. coli* H9049 by binding bacterial siderophores and reducing the availability of iron during infection of mice (Flo et al., 2004). Interestingly, it has been suggested that lipocalin-2 selectively restricts the growth of pathogenic *E. coli* and provides an advantage to commensal *E. coli* during infection by the pathogen (Singh et al., 2015). Similar to results observed with lipocalin-2, host lactoferrin is also involved in restricting iron; lactoferrin helped protect mice against infection by *Mycobacterium tuberculosis* (Schaible et al., 2002) and in some instances reduced granuloma severity (Welsh et al., 2010). In humans, deficiencies in iron regulation, such as hemochromatosis have been correlated with a higher risk of infection and increased complications (Gerhard et al., 2001; Höpfner et al., 2001). This effect was even observed during oral iron supplementation in populations with wide-spread iron deficiency, as treated groups experienced increased infections by enteric pathogens (Cross et al., 2015) These findings emphasize the importance of keeping iron availability low to inhibit pathogen colonization and growth.

While many hosts have evolved methods for sequestering iron to inhibit bacterial growth, pathogenic bacteria have also evolved mechanisms to circumvent these host responses. Since exogenous free-iron is often limited in the environment, Gram-negative bacteria in particular often have outer membrane receptors for ferrous iron (Kammler et al., 1993). The FeoB protein from *E. coli* binds and transports ferrous iron across the outer membrane and FeoB homologs have been identified in several bacterial pathogens, including *Campylobacter jejuni* (Naikare et al., 2006). Additionally, several pathogenic bacteria also secrete molecules called siderophores, which bind ferrous iron with high affinity and transport it back to the bacterium. Similarly, pathogens can also secrete hemophores, which bind to iron-containing compounds, such as heme or hemoglobin and transport them into the cell (Holden et al., 2016; Ellermann and Arthur, 2017; Saha et al., 2017). In addition, some Gram-negative pathogens do not produce their own siderophores or hemophores, but instead encode outer membrane receptors that bind to siderophores produced by other organisms. Referred to as xenosiderophores, molecules such as enterobactin are synthesized by other bacterial species, but can be utilized by bacterial pathogens such as *C. jejuni* (Xu et al., 2010; Pletzer et al., 2016).

In addition to the above methods for iron acquisition, some Gram-negative pathogens have evolved the ability to utilize host molecules to obtain iron (Lindgren et al., 2015; Smith and Wilks, 2015; Balhesteros et al., 2017). The focus of this review is the use of heme as an alternative iron source for these pathogens. The use of heme as an iron source is beneficial for several reasons, including: (i) it is the most abundant source of iron within the host environment; (ii) not all organisms are able to use every form of iron acquisition mentioned above, e.g., *C. jejuni* is unable to produce its own siderophores (Naikare et al., 2013); (iii) the use of heme allows the pathogen to acquire iron while the host attempts to restrict it; and (iv) some Gram-negative pathogens, termed heme auxotrophs, lack the ability to synthesize heme and must acquire it for their own cellular processes from the environment, e.g., *Haemophilus influenzae* (Choby and Skaar, 2016; Zambolin et al., 2016; Hardison et al., 2018). For these reasons, it is important to understand how heme acquisition systems work in Gram-negative pathogens.

### Release of Hemoglobin Bound Heme From Erythrocytes

In humans, the hemoglobin contained in erythrocytes is an abundant source of iron. Hemoglobin is a tetrameric protein that contains four heme subunits, with each binding an iron molecule (Perutz et al., 1960); heme has an affinity for histidine and these residues account for heme binding and the quaternary structure of hemoglobin (Kakar et al., 2010). While hemoglobin is required for transporting oxygen in humans, heme-bound hemoglobin can also be a source of iron for invading pathogens and can be released through cell lysis (Martins et al., 2016). Hemolysis can be caused by multiple factors that disrupt the cellular membrane. These include exposure to toxins that target the phospholipids in the erythrocyte membrane or creation of membrane pores (Brok et al., 1994; Linhartová et al., 2010). A major category of pore-forming toxins is the RTX family. While these toxins may not solely target erythrocytes, many of them have been associated with hemolysis and are characterized by glycine and aspartate repeat regions that bind calcium and single-step secretion across the cell membrane (Linhartová et al., 2010).

The best-studied RTX hemolysin is HlyA, which is secreted by *E. coli*. HlyA is a 107 kDa protein (Sánchez-Magraner et al., 2007) that contains an N-terminal amphipathic region that allows for insertion into the erythrocyte cell membrane (Schindel et al., 2001) and a C-terminal domain containing RTX repeats and a calcium binding region (Sánchez-Magraner et al., 2007). When the C-terminal domain is bound by calcium, HlyA experiences a conformational change that makes the beta sheet region more compact, so that when the protein inserts into the cell membrane, a pore is created that disrupts the ion equilibrium within the cell (Schindel et al., 2001). Experiments have shown that erythrocytes exposed to 100 HU/ml of HlyA experienced an outflow of potassium ions and influx of calcium ions, leading to cell lysis (Bhakdi et al., 1986).

In addition to RTX cytolysins, phospholipases have been speculated to induce hemolysis (Brok et al., 1994). These proteins target the lipids of cells and may disrupt the membrane to induce cell lysis; however, their role in hemolysis is inconclusive and the exact mechanisms are not well-understood (Grant et al., 1997; Reimann et al., 2016). For example, phospholipase A from *Campylobacter coli* was found to induce hemolysis, but its role during infection has not been examined (Grant et al., 1997). Subsequent work found that PldA in *E. coli* plays an important role in maintaining the symmetry of the outer membrane by removing phospholipids that accumulate under stressful conditions (Reimann et al., 2016), raising the question as to whether PldA specifically causes hemolysis.

While the exact mechanisms may not be completely understood, hemolysins have been linked to increased severity and hemorrhagic infection. In uropathogenic *E. coli* (UPEC), increased expression of HlyA resulted in increased inflammation and disease severity in a murine model (Nagamatsu et al., 2015). As expected, when an *hlyA* mutant was introduced to a murine model, HlyA production, macrophage cell death, and bladder bleeding were all reduced, indicating that these systems are important for virulence (MV Murthy et al., 2018).

### Transportation of Heme Using Hemophores

After hemoglobin is released from erythrocytes, the bacterial cell must be able to uptake the heme from the host environment. Gram-negative bacteria have been shown to either secrete proteins that bind heme and bring it to a receptor on the cell surface or simply encode the receptor and directly bind heme itself (Lindgren et al., 2015; Smith and Wilks, 2015; Balhesteros et al., 2017; Kawano et al., 2018). These secreted proteins, known as hemophores, have been identified in several pathogenic bacteria and may play an important role in pathogenicity. For example, *Porphyromonas gingivalis* was grown in the presence of macrophages (THP-1) with heme as an iron source (Olczak et al., 2015). The number of attached and internalized cells were compared between wild-type *P. gingivalis* and a hemophore mutant over a 20-h period, finding that the mutant exhibited decreased growth and invasion of the macrophages (Olczak et al., 2015).

A major class of hemophores are the HasA proteins, which have been identified in multiple bacterial pathogens, including *Serratia marcescens* and *Pseudomonas aeruginosa* (Yukl et al., 2010; Kumar et al., 2016; Dent et al., 2018). Substantial research has been conducted on the structure of HasA (Izadi et al., 1997; Jepkorir et al., 2010), which has found that the protein exists as a 19 kDa monomer with a one-to-one HasA-to-heme binding ratio (Izadi et al., 1997). To understand the conformational changes that occur when HasA binds to heme, the crystal structures of both unbound and bound HasA have been solved. Once bound to heme, HasA binds to HasR, resulting in the transfer of heme to HasR (Izadi-Pruneyre et al., 2006; Dent et al., 2018). While it is not clear what structural changes occur when HasA transfers heme, it has been shown that HasA can transfer heme to HasR *in vitro* when ATP is absent, indicating that this transfer does not require energy (Izadi-Pruneyre et al., 2006).

### Heme Transfer Across the Outer Membrane

**Figure 1:**
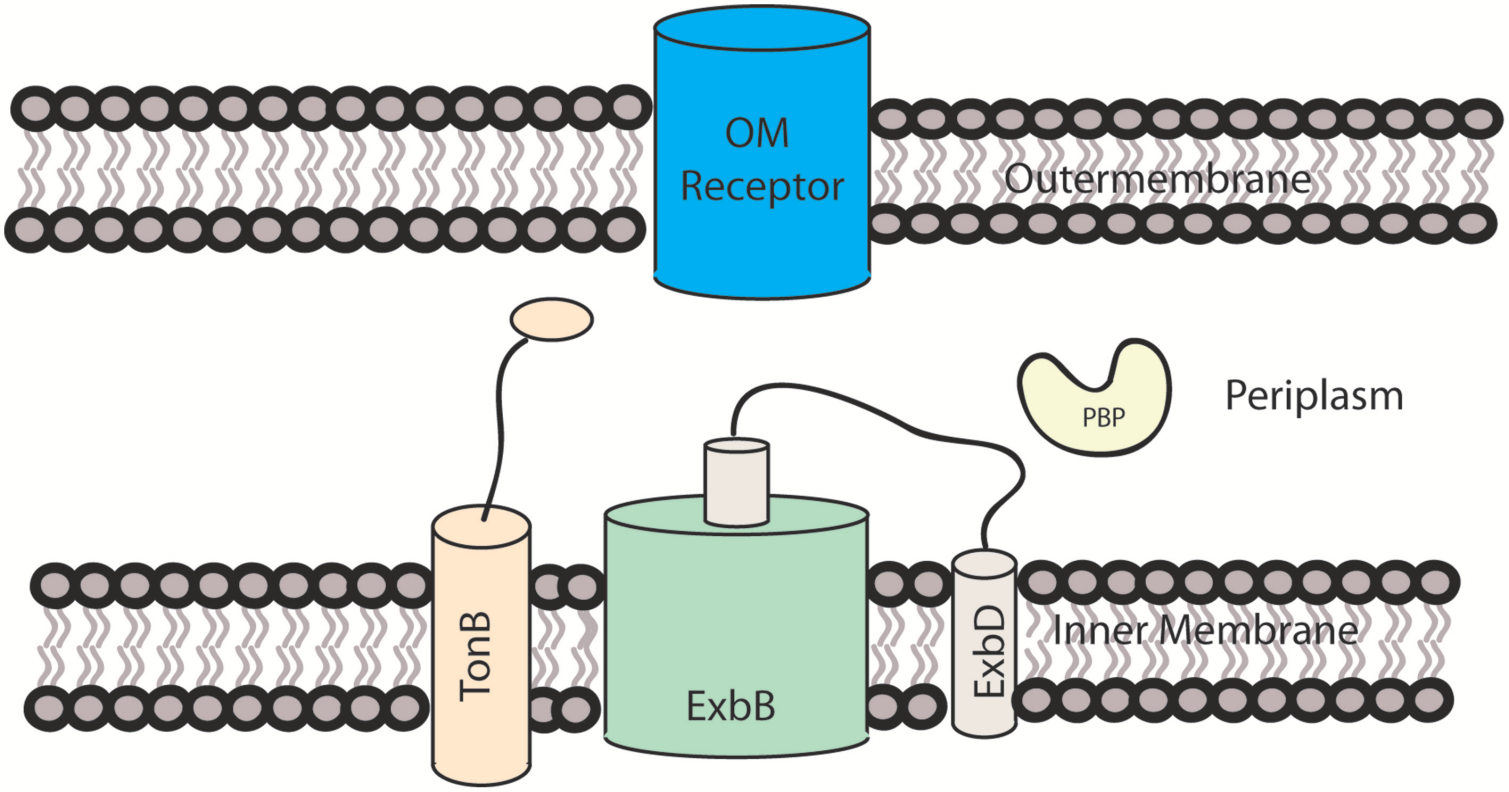
Heme transport across the outer membrane. The TonB-ExbB-ExbD is located in the inner membrane and utilizes a proton motive force to transfer energy required for heme transport to the receptor located in the outer membrane.

Since Gram-negative bacteria have two membranes, they have evolved mechanisms to overcome the energy barrier of transporting large molecules across both membranes. For the transport of heme across the double membrane and into the periplasmic region, Gram-negative bacteria most commonly use a mechanism involving an outer membrane receptor and a TonB-ExbB-ExbD complex in the inner membrane (Celia et al., 2016). An example of this can be seen in Figure 1. This process has been best characterized in *E. coli*, but has also been identified in other Gram-negative bacteria. For example, comparison of the heme receptors, HmbR and HemR from *N. meningitidis* and *Y. enterocolitica*, with other TonB-dependent outer membrane receptors, found that residues His128 and His 461 along with FRAP and NPNL amino acid motifs, were conserved along with the overall beta barrel that spans the outer membrane (Bracken et al., 1999). The lack of conservation of His128 and His461 across non-heme specific TonB receptors indicates that, like in hemoglobin, these residues are involved in heme binding.

While some outer membrane (OM) heme receptors use hemophores like HasR, many heme receptors bind free heme or heme bound to protein complexes, such as hemoglobin or hemopexin. Examples of these proteins are FrpB1 from *Helicobacter pylori* and ChuA from *C. jejuni*, which have been shown to bind to heme and/or hemoglobin using UV-VIS spectrophotometry (Ridley et al., 2006; Carrizo-Chávez et al., 2012). Because passive release is possible, this is an important step for determining if the receptor is binding to the hemoglobin or heme that has been already released from hemoglobin. For this reason, growth assays with bacterial strains in the presence of hemoglobin are not sufficient to determine if the receptor can bind to hemoglobin. However, further research must be conducted to determine the exact structure and interactions between these outer membrane receptors with heme. In addition, Gram-negative bacteria may have more than one receptor for binding to extracellular heme. In *P. aeruginosa*, PhuR has been identified along with HasR as being required for heme utilization, potentially through heme uptake (Smith et al., 2015). While in some bacteria, the presence of multiple receptors may be redundant, the primary functions of HasR and PhuR are to sense heme and uptake heme, respectively (Smith and Wilks, 2015). This system is important because it indicates having different receptors for similar functions may aid in pathogen survival and is not always redundant.

Once heme is bound to the outer membrane receptor, energy is required to transport heme across the outer membrane. While the total energy requirement has not been verified, a proton motive force is created by the transportation of protons from the periplasmic space to the cytoplasm (Celia et al., 2016). ExbB facilitates this through its alpha-helical structure that spans the inner membrane. The helices form a pentamer pore in which one of the helices of ExbD resides. Because the size of ExbD is ~140 amino acids (WP_013364771.1), it is not possible for the entire protein to reside within the pore. It is therefore speculated that the remaining hydrophobic helices are located in the membrane outside of ExbB (Celia et al., 2016), as demonstrated in Figure 1. It is not known how the proton motive force is transformed into energy for the transport of heme across the membrane, but two mechanisms have been proposed, with ExbD either rotating or acting like a piston, with ExbD moving along ExbB's central axis. This mechanical motion is believed to interact with TonB, causing it to interact with the outer membrane receptor (Celia et al., 2016). The C-terminal domain of TonB interacts with the TonB box, a disordered region at the N-terminus of the OM receptor in the periplasm of the cell, which may be responsible for the energy transfer from the TonB complex to the OM heme receptor (Shultis et al., 2006).

A protein performing a more specific function, but with similar structure to TonB, has been identified in *Serratia marcescens*. This protein, HasB, is encoded by the *has* operon, which also encodes HasA and HasR, mentioned above (Paquelin et al., 2001). While the structure of the entire protein has not been determined, an additional alpha-helix not seen in TonB has been identified in the C-terminal domain of HasB (de Amorim et al., 2013). Using isothermal titration calorimetry, it has been shown that the C-terminal domain of HasB, like TonB, interacts with the periplasmic domain of HasR. Studies with a second heme receptor, HemR, of *S. marcescens* indicate that HasB cannot replace TonB for other outer membrane receptors because of its specificity for HasR (Benevides-Matos et al., 2008). Overall, the discovery of a TonB-like protein is significant in that it shows that Gram-negative bacteria may have multiple receptors and systems that enable it to acquire heme.

### Inner Membrane Transport

Transport of heme across the inner membrane is energy dependent and is often facilitated by an ATP-binding cassette (ABC) transporter and a periplasmic binding protein (PBP) (Qasem-Abdullah et al., 2017). The PBP is responsible for transporting the heme between the two membranes and to the ABC transporter. The first crystal structures of a PBP were solved for ShuT in *Shigella dysenteriae* and PhuT in *Pseudomonas aeruginosa*, which had a structure similar to the vitamin B_12_ PBP, BtuF (Ho et al., 2007). While the initial study of the crystal structures indicated that little change occurred with heme binding, molecular simulations indicated that the proteins may undergo an opening and closing motion when the C and N termini bind heme (Ho et al., 2007; Liu et al., 2008). A recent study that solved the crystal structure of BhuT, a PBP from *Burkholderiae cenocepacia*, agreed with this molecular simulation since the protein was in an “open state” without heme, but closed when bound in a one-to-one ratio (Naoe et al., 2017).

Heme PBPs have also been identified in *Vibrio cholerae* (HutB) and *Yersinia pestis* (HmuT) (Mattle et al., 2010; Agarwal et al., 2015). While most of the discovered PBPs bind to heme in a ratio of 1:1, HmuT has been found to bind two heme molecules simultaneously (Mattle et al., 2010). Similar to other PBPs, HmuT has two beta sheet domains connected by an alpha helix; however, HmuT also has an increased distance between residues Y70 and R167 when compared to other PBPs. Along with a histidine residue present in the heme binding pocket, these findings may explain HmuT's ability to bind more than one heme molecule.

**Figure 2:**
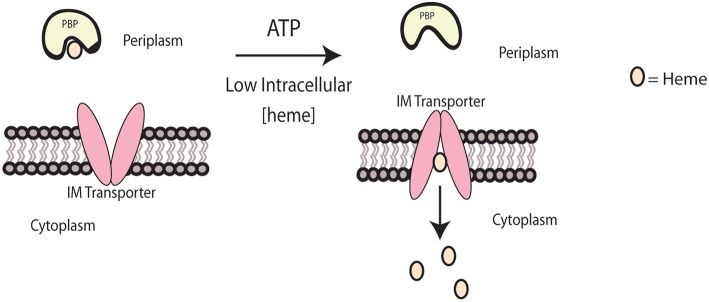
Heme transport across the inner membrane. Heme transport across the inner membrane is modeled for *Yersinia pestis*. When intracellular heme concentrations are low, the IM transporter, HmuUV, will transport heme from the PBP, HmuT, across the inner membrane into the cytoplasm.

For heme to be shuttled across the inner membrane once the PBP binds to the ABC transporter, ATP is required. The general mechanism of ABC transport involves the binding of ATP to the transporter and hydrolysis, which initiates a conformational change that enables heme transport and the release of the PBP from the transporter (Qasem-Abdullah et al., 2017). In *Yersinia pestis*, HmuUV has been identified as a Type II ABC transporter—a complex that can transport large molecules like heme, as opposed to the smaller ions and amino acids that are transferred by Type I transporters (Woo et al., 2012; Qasem-Abdullah et al., 2017). HmuUV contains a region of alpha helices that are believed to form an opening for heme transport across the inner membrane (Woo et al., 2012). The crystal structure for this complex has been determined when bound to the PBP, HmuT, and to ATP. In this complex, HmuUV's channel is open to the periplasm, but shifts its opening to the cytoplasm upon binding by HmuT. This action would allow for the transfer of two heme molecules at one time (Mattle et al., 2010), which is shown in Figure 2. Comparison of HmuUV to other complexes demonstrated similarity to the vitamin B_12_ transporter, BtuCD, but still exhibited differences in terms of binding affinities with the PBP and its dependence on ATP (Qasem-Abdullah et al., 2017).

Additional heme ABC transporters have been identified in the Gram-negative pathogens, *Shigella dysenteriae* (ShuUV) (Burkhard and Wilks, 2008) and *Burkholderia cenocepacia* (BhuUV) (Naoe et al., 2016). Like HmuUV, ShuUV has been shown to transport heme using proteoliposomes and hydrolyze ATP (Burkhard and Wilks, 2008). The crystal structure of BhuUV was found to contain slight differences when compared to HmuUV, including a change in the direction of the pore opening without the PBP present (Naoe et al., 2016). Additionally, bacteria may have transporters that are not specific to heme or encode multiple ABC transporters. One example includes the HmuR heme transporter in *Burkholderia thailandensis*, which also transports zinc during oxidative stress (Si et al., 2017). Redundancy was also suggested in a *C. jejuni* growth study where mutants lacking the *chuBCD* genes, the predicted ABC transporter, were still able to grow using heme or hemoglobin as an iron source (Ridley et al., 2006). This result indicated that the receptor can use a transporter that is not dedicated to its import of heme. As a result, much work is still needed to determine how heme interacts with the PBP and is transported across the inner membrane by these systems in Gram-negative pathogens.

### Release of Iron From Heme

As mentioned previously, heme is an abundant source of iron in the human body and is primarily associated with erythrocytes. Since erythrocytes regularly become senescent, the host needs to degrade heme to recover iron and to reduce the possibility of toxic free radical formation (Li and Stocker, 2009). This may be an evolutionarily ancient process, since some bacteria use similar enzymes to release iron from heme that has been trafficked to the intracellular space. These enzymes belong to one of three distinct families of heme oxygenases: HO-1 heme oxygenases, ChuZ heme oxygenases, and IsdG/I heme oxygenases, although it is unclear whether all are present in Gram-negative bacteria.

#### HO-1 Heme Oxygenases

The canonical bacterial HO-1 heme oxygenases are structural and functional homologs of mammalian oxygenases (Wilks and Heinzl, 2014). These enzymes consist of alpha helices that create a fold that comprises the heme-binding pocket (Schuller et al., 2001); upon binding heme, the distal and proximal helices bend around the bound heme (Unno et al., 2007; Wilks and Heinzl, 2014). The distal helix binds heme via glycine residues that are conserved across many HO-1s (Schuller et al., 1999) while the proximal helix contains a conserved histidine residue that interacts with the heme. The potential exists that multiple heme orientations occur within the binding pocket, depending on interactions with water molecules and the side chains of the oxygenase (Schuller et al., 2001). While there are some differences in the distal helix between bacterial and mammalian HO-1 enzymes, the mechanisms of heme degradation appear to be similar.

Even though various molecules were found to be involved in heme utilization and degradation, only recently has the exact mechanism been determined. Research on mammalian HO-1 heme oxygenases revealed that three major steps occur to release iron from heme and that oxygen is required to convert the heme to the final products of free ferrous iron, biliverdin, and carbon monoxide (Matsui et al., 2010). In the first step, heme is catalyzed to ferric-hydroperoxide and then alpha-meso-hydroxyheme through the addition of electrons and oxygen molecules (Matsui et al., 2010; Wilks and Heinzl, 2014). The second step involves the formation of verdoheme and carbon monoxide from alpha-meso-hydroxyheme (Matsui et al., 2010). Because this step can occur without the aid of a catalyst, the presence of carbon monoxide was found to be a poor indicator of whether a protein is a heme oxygenase. In the third step, the heme oxygenase can utilize either oxygen or hydrogen peroxide to catalyze the opening of the verdoheme ring to form biliverdin (Matsui et al., 2005), releasing iron following the reduction of ferric iron to ferrous iron (Matsui et al., 2010). Although this mechanistic understanding advanced the field, details including the location of oxygen binding in step two remain unknown.

#### ChuZ Heme Oxygenases

Recently, heme oxygenases from epsilonproteobacteria were found to belong to a unique family whose folds differ from those of the HO-1 oxygenases. For example, ChuZ from *Campylobacter jejuni* has been shown to bind heme in a similar manner as the HO-1 oxygenases, but the binding site located at the interface of the two dimers is not as rigid as that of the HO-1 heme oxygenases (Ridley et al., 2006; Zhang et al., 2011). Additionally, the dimers of ChuZ contain a distinct split barrel region in the C-terminal domain (Zhang et al., 2011) and the protein may contain another heme binding site on the surface of ChuZ. This additional binding site is similar to an enzyme that binds and stores heme in *Haemophilus ducreyi*, suggesting that ChuZ may also play a role in reducing heme toxicity by binding excess heme. Comparison of ChuZ amino acid sequence to HugZ in *H. pylori* provided evidence that HugZ may also belong to the same family, but a similar secondary heme binding site has not yet been demonstrated for HugZ (Guo et al., 2008; Zhang et al., 2011). Examples are outlined in Table 1.

**Table 1:** Heme-degrading proteins.

**Protein**	**Organism**	**Products**	**Characteristics**	**References**
HmuO	*Corynebacterium diphtheriae Neisseria meningitidis*	Biliverdin Carbon Monoxide	• Similar structure to mammalian HO	Schuller et al., 2001; Kunkle and Schmitt, 2007; Matsui et al., 2010
IsdG	*Staphylococcus aureus*	Staphylobilin Formaldehyde	• Found primarily in gram positive bacteria • Heme ruffling	Reniere et al., 2010
MhuD	*Mycobacterium tuberculosis*	Mycobilin	• Heme ruffling	Nambu et al., 2013
ChuZ	*Campylobacter jejuni Helicobacter pylori* (*Hug Z)*	Biliverdin Carbon Monoxide	• Split barrel region in C domain of dimers • Two heme binding sites	Ridley et al., 2006; Guo et al., 2008; Zhang et al., 2011
ChuW	*Escherichia coli* O157:H7	Anerobilin	• Degrades heme without oxygen	LaMattina et al., 2016

#### IsdG/I Heme Oxygenases

IsdG/I heme oxygenases are named for the well-studied oxygenases in *Staphylococcus aureus*. While these enzymes perform the same general function of heme degradation, their unique structure and mechanisms make them distinct from the HO-1 and ChuZ enzyme families. IsdG from *S. aureus* consists of two monomers that form beta sheets, as opposed to the alpha helices seen in the HO-1 family (Wu et al., 2005). Additionally, it was observed that oxygen was in a different location in the active site of IsdG and the presence of water was not required in the binding site. This led to the hypothesis that the product of IsdG/I oxygenases is different from that of the HO-1 family (Reniere et al., 2010). By NMR, it was also found that the molecular weight of the final product was different than that of biliverdin or bilirubin. Combined with the difference in color of the solid (red-orange as opposed to blue-green), the final product was determined to be staphylobilin, a different chromophore of bilirubin. In addition to the production of staphylobilin, formaldehyde instead of carbon monoxide was found to be produced as a byproduct (Matsui et al., 2013).

Putative oxygenases in other organisms have been found to contain the same beta barrel structure of the IsdG/I family, including the predicted IsdG homolog MhuD in *Mycobacterium tuberculosis* (Chim et al., 2010; Ahmed et al., 2016). However, differences in the three-dimensional structure were detected enabling MhuD to bind two heme molecules as opposed to the one-to-one binding that occurs with IsdG. Interestingly, the binding of the two heme molecules to MhuD is enzymatically inactive as the heme is not degraded. Similar to IsdG, the reaction catalyzed by MhuD bound to a single heme molecule does not produce carbon monoxide or biliverdin and instead produces mycobilin (Nambu et al., 2013). One reason for this difference may be the structure of heme when bound to MhuD. Instead of retaining its planar structure, heme becomes non-planar in a process termed ruffling (Matsui et al., 2013). This alternate orientation may account for the retention of carbon at the cleavage site, producing mycobilin without any carbon monoxide production.

It was thought previously that the IsdG family of oxygenases were found primarily in Gram-positive bacteria; however, heme oxygenases similar to IsdG have been identified in the algae *Chlamydomonas reinhardtii* (Lojek et al., 2017) and in the Gram-negative bacterium *Bradyrhizobium japonicum* (Puri and O'Brian, 2006). This latter oxygenase appears to be more similar in sequence to IsdG from *S. aureus* than the heme oxygenases belonging to the HO-1 family. Despite this sequence similarity, the structure and function of the protein more closely resemble that of the HO-1 family, including the production of biliverdin (Puri and O'Brian, 2006). Consequently, it remains unclear whether the enzymes from *B. japonicum* exhibit enough sequence similarity to belong to the IsdG family or whether the classification of newly identified heme oxygenases should rely on structure and function. If these enzymes truly belong to the IsdG family, these findings suggest that this family of heme oxygenases is not limited to Gram-positive bacteria, but may also include Gram-negative bacteria and eukaryotes.

#### Heme Degradation Without Oxygen

It has recently been shown that iron can be released from heme without the use of oxygen. An example of this is ChuW, a *S*-adenosylmethionine methyltransferase in pathogenic enterohemorrhagic *E. coli* O157:H7 (EHEC) (LaMattina et al., 2016). ChuW can degrade heme in the absence of oxygen using alternate electron donors, such as flavodoxin and produces anaerobilin as opposed to biliverdin. Such a mechanism is predicted to benefit enteric pathogens like EHEC since they have a readily available source of heme during infection, but must be able to liberate the iron in an anaerobic environment. Since the importance of this system remains to be demonstrated in an infection model, more research is needed to determine how anaerobic heme degradation may aid pathogenic bacterial species.

### Regulation of Heme Utilization Systems

As mentioned above, several pathogens use heme as a source of iron during colonization and infection, but excess quantities of heme and iron must be avoided since they can be toxic to bacterial cells (Anzaldi and Skaar, 2010). To prevent excess iron, bacteria encode finely-tuned regulatory mechanisms that control iron and iron-containing protein import into the cell. As a result, these regulatory networks are incredibly important to infection since pathogens must bring in iron to combat nutritional immunity while maintaining cell viability. While some of these regulatory systems have been studied, such as the examples outlined in Table 2, much remains unknown about the specific mechanisms utilized by pathogenic bacteria for regulating heme uptake.

**Table 2:** Examples of heme regulatory systems in Gram-negative bacteria.

**Regulation type**	**Examples**	**References**
Transcriptional	Fur	van Vliet et al., 2002; Butcher et al., 2012; Visca and Imperi, 2018
Transcriptional co-regulation	• Fur + HeuR • Fur + HupR • Fur + sRNAs	Litwin and Quackenbush, 2001; Ridley et al., 2006; Porcheron and Dozois, 2015; Johnson et al., 2016; Reinhart et al., 2017; Tronnet et al., 2017
Post-transcriptional	• Biliverdin regulation of *hasA* in *P. aeruginosa* • RNA thermometer regulation of *shuA* (*S. dysenteriae*) and *chuA* (*E. coli*)	Kouse et al., 2013; Mouriño et al., 2016; Dent et al., 2018
Post-translational	PhuS/HemO in *P. aeruginosa*	O'Neill et al., 2012
Indirect	Free Diffusion of Heme in pilQ mutant sRNAs	Chen et al., 2004; Anzaldi and Skaar, 2010

**Figure 3:**
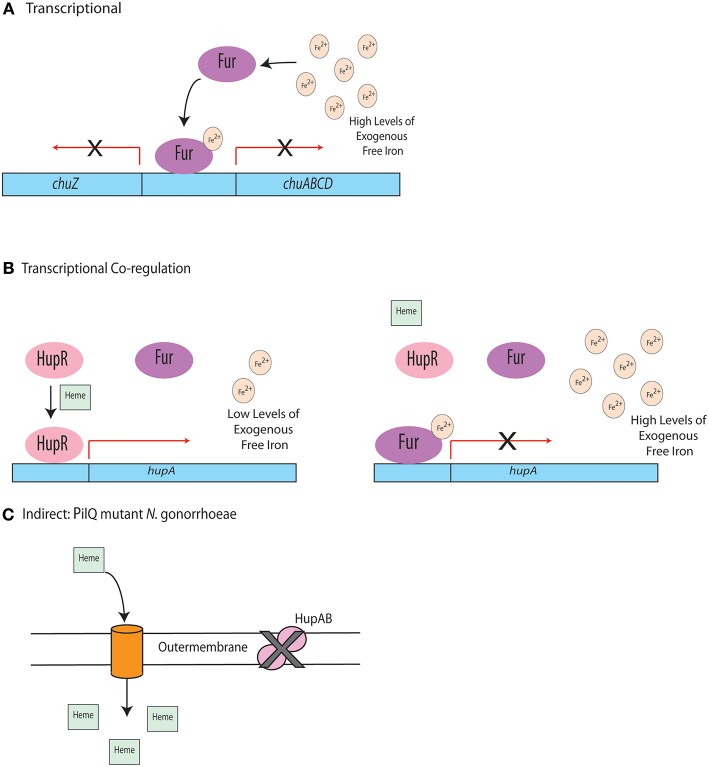
Regulation of heme uptake systems. **(A)** In *C. jejuni*, under high iron conditions, Fur binds to the promoter region of the *chu* operon, repressing transcription. **(B)** When heme is present and free iron concentrations are low, HupR in *Vibrio vulnificus* binds to the promoter of *hupA* and the outer membrane heme receptor is transcribed. Transcription is repressed by Fur in the presence of high levels of free iron even if heme is present. **(C)** Indirect regulation of heme uptake in a PilQ mutant.

One regulatory system observed widely across Gram-negative bacteria is Fur-dependent regulation. While it has been best studied in *E. coli*, Fur has been identified in other Gram-negative pathogens, including *H. pylori, C. jejuni*, and *P. aeruginosa* (van Vliet et al., 2002; Butcher et al., 2012; Visca and Imperi, 2018). While Fur can regulate multiple systems, it is usually involved in controlling the acquisition of iron and/or other metals (Mey et al., 2005; Seo et al., 2014). Canonically, Fur is a repressor that binds to the promoter region of many genes when iron availability is high, using iron (II) as a cofactor (van Vliet et al., 1998) to negatively regulate the transcription of those genes (Troxell and Hassan, 2013; Fillat, 2014). Examples of the regulatory role of Fur are outlined in Figure 3A.

In the pathogen *C. jejuni*, Fur was found to regulate the expression of the heme receptor, ChuA, and the enterobactin receptor, CfrA. While the role of *C. jejuni* Fur in disease remains unknown, Fur was found to be required for efficient colonization of infant mice by the Gram-negative pathogen *Vibrio cholera* (Mey et al., 2005). Whether this was due to dysregulated iron homeostasis remains unknown, since Fur was also shown to regulate systems involved in biofilm formation in *P. aeruginosa* (Wiens et al., 2014). This may be due to the observation that Fur often contains binding sites for other metals, including zinc (Butcher et al., 2012) and cobalt (Dian et al., 2011). As a result, research is being conducted to fully define the broad role of Fur in both metal acquisition and host infection. In addition to Fur regulation, other regulatory proteins have been found that bind to the same promoters as Fur, indicating co-regulation of some cellular systems. In *C. jejuni*, a PAS-domain containing regulatory protein, termed HeuR, was found to positively regulate the heme utilization system and also bind to a promoter fragment that contains putative Fur binding sites (Ridley et al., 2006; Johnson et al., 2016). This regulator which is specific to the *Campylobacter* genus, did not impact transcription of Fur, indicating that it may work with Fur to control heme utilization in *C. jejuni*. Interestingly, expression of the heme utilization system in *C. jejuni* is up-regulated during human infections, indicating this this regulatory system may be particularly important to infection (Crofts et al., 2018).

There is precedence for co-regulation of heme utilization systems. In *Vibrio vulnificus*, competition between Fur and the positive regulator, HupR, has been observed (Litwin and Quackenbush, 2001). In low iron environments, when heme is present, HupR can bind to the promoter of *hupA*, the gene that encodes the outer membrane heme receptor, and induce expression. In high iron conditions, Fur becomes an active repressor and binds to the *hupA* promoter, preventing HupR binding (Litwin and Quackenbush, 2001). This regulatory mechanism is depicted in Figure 3B. While this study demonstrates a relationship between two regulatory proteins, the exact mechanism of how they positively and negatively impact *hupA* expression remains unknown. The study of Fur in these systems is further complicated by observations that Fur binds to promoter sites of PerR, a regulatory protein that responds to oxidative stress, indicating that Fur may play a complex role in cellular responses to stress (Holmes et al., 2005).

Fur has also been found to act along with sRNAs, such as RhyB in *E. coli* (Porcheron and Dozois, 2015; Tronnet et al., 2017) and PrrF in *P. aeruginosa* (Reinhart et al., 2017), to directly or indirectly regulate intracellular iron levels. In *P. aeruginosa*, PrrF negatively impacts the transcription and resulting translation of proteins that bind iron in order to reduce iron requirements by the organism (Reinhart et al., 2017). Utilizing a murine lung infection model, Reinhart et al., demonstrated the regulatory role of PrrF in *P. aeruginosa* during colonization of a host. Mutants lacking functional PrrF sRNAs were unable to colonize efficiently, although the exact mechanisms resulting in this colonization deficiency are not well-understood (Reinhart et al., 2017). All together, these studies indicate a complex role of Fur in the regulation of heme and iron uptake systems. While Fur in some organisms, such as *E. coli* has been studied more extensively, much remains unknown regarding Fur regulation in other Gram-negative pathogens and its impact on their ability to colonize susceptible hosts.

An interesting example of indirect heme regulation has been identified in *Neisseria gonorrhoeae*. This regulatory mechanism involves a point mutation in the *pilQ* gene, which results in a loss of function for the HpuAB heme uptake system in *N. gonorrhoeae* (Chen et al., 2004). Although this uptake system is non-functional, this point mutation results in the ability to utilize PilQ to form pores and allow for diffusion of heme across the membrane via PilT (Anzaldi and Skaar, 2010). This process is outlined in Figure 3C. Similar mechanisms for indirect iron and heme regulation may occur in other pathogenic organisms, but have not been identified thus far. Regardless, elucidating such mechanisms would provide insight into the complex mechanisms by which Gram-negative bacterial pathogens regulate heme uptake when colonizing the host.

While transcriptional regulatory systems, such as those described above have been identified in diverse Gram-negative bacterial species, post-transcriptional regulation remains more elusive. A proposed mechanism for post-transcriptional heme regulation has been described for the *hasA* system in *Pseudomonas aeruginosa*. In this system, products of heme degradation, such as biliverdin (BVIXβ/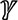), act as post-transcriptional regulators for heme uptake—although the exact mechanism of regulation remains unknown (Mouriño et al., 2016; Dent et al., 2018). Another method of post-transcriptional regulation of heme utilization has been described in *Shigella dysenteriae* and pathogenic *E. coli*. The heme receptors, *shuA* in *S. dysenteriae* and *chuA* in *E. coli*, are regulated post-transcriptionally in a temperature-dependent manner by an RNA thermometer (Kouse et al., 2013) where, depending on the temperature, these organisms can post-transcriptionally regulate the genes involved in heme binding.

In addition to post-transcriptional regulation, post-translational control of heme in gram negative pathogens remains ill-defined. In *P. aeruginosa*, PhuS transports heme to the heme oxygenase, HemO (O'Neill et al., 2012). The equilibrium between heme-bound and unbound PhuS determines how heme is transported by the pathogen. Additionally, mutants have indicated that HemO influences heme uptake and its expression is repressed by iron (O'Neill et al., 2012). Overall, post-translational regulation of heme utilization has not been well-studied in Gram-negative pathogens, but would make for interesting targets of future research.

## Conclusion

Since iron is required for growth by many bacteria, animal hosts utilize multiple iron sequestering molecules to limit free-iron availability and combat pathogen colonization. As a result, free-iron is limited within the host, leading many pathogens to develop various strategies to obtain iron from host-derived molecules, like heme, during infection. Much of the research in this area has focused on how Gram-positive pathogens, especially *S. aureus*, acquire iron from these sources; however, it is becoming increasingly apparent that Gram-negative bacteria can also acquire iron from heme during infection. Due to divergence, the systems in Gram-negative bacteria will likely differ from those of Gram-positive pathogens, especially in the case of heme utilization since it requires the transport of heme across the bacterial outer membrane. While we have begun to understand the structure and mechanisms of proteins involved in heme uptake and utilization in pathogens like *E. coli* and *Yersinia pestis*, much still remains unknown about lesser studied organisms like *C. jejuni* and *H. pylori*. While homologous proteins have been identified for heme uptake and oxygenation in these lesser studied organisms, differences in protein sequence and predicted structure indicate that the complexes may assemble and operate differently. This makes it particularly important to study these systems in diverse organisms, since a comprehensive understanding will be necessary for the development of these as targets for new therapies. Since many Gram-negative pathogens are becoming increasingly resistant to antibiotics, identifying and characterizing these potential drug targets is of increasing importance to human health.

## Author Contributions

All authors listed have made a substantial, direct and intellectual contribution to the work, and approved it for publication.

### Conflict of Interest Statement

The authors declare that the research was conducted in the absence of any commercial or financial relationships that could be construed as a potential conflict of interest.
